# On the Climatic Conditions of the Summer of 1853, Most Directly Affecting Its Sanatary Character

**Published:** 1853-12

**Authors:** 


### ECLECTIC DEPARTMENT,

### AND SPIRIT OF THE MEDICAL PERIODICAL PRESS.

---

[*Note Editorial.*—It had been our intention to prepare an abstract of this article, with some additional statements, in reference to the health of the city of Buffalo during the past summer.

Two circumstances have conspired to prevent this: We were unable to procure the necessary hygrometric tables, none such having been kept during the past year in this locality. Secondly, the mortuary returns of the city are very inaccurate. The first difficulty will hereafter be obviated by the kindness of a scientific gentleman who has consented to make the necessary observations; while for the other, we must trust to the sextons' reports of burials, which, though inaccurate as to number of deaths, will present a fair average of the causes of mortality.] S. B. H.

*On the Climatic Conditions of the Summer of* 1853, *most directly affecting its Sanatary Character.* (A Report to the Secretary of the Smithsonian Institution, Washington.) By Lorin Blodget, Esq., in charge of the Meteorological Department.

The most important condition of our summer climate affecting its sanatary character is the most difficult to measure. Humidity, or the proportion of the amount of moisture required to fully saturate the air, which it is found to contain at any time, is, probably, the controlling sanatary condition at all high temperatures. This is of course exclusive of local or miasmatic causes, which are climatic only in the more limited sense, and secondary to temperature and humidity under all circumstances.

The determination of humidity in such a manner as to make the results comparable at different places, and available for general grouping, is the most recent point gained in meteorological observation. Without concert of action, and uniformity in the manner of observation, and in the instruments used, the earlier observations served only for comparisons at the same station, and even that imperfectly. The various forms of hygrometer and dew-point instruments have been generally superseded by the wet-bulb thermometer. The observation of the *temperature of evaporation*, as given by this instrument, under circumstances which truly represent the mass of surface atmosphere at the time, or what may be termed normal conditions in respect to the *evaporating effect*, has entire uniformity of character, and such observations may be relied upon for the fullest comparison and illustration.

The summer of 1853 has been extraordinary in its climatic conditions, and the extremes of temperature and humidity were much more striking and clearly defined than usual. The relation which these conditions may have to the general sanatary character of a month or season, may therefore be analyzed under unusually favorable circumstances, if our observations are sufficient to define them with precision. The purpose of this paper is to present them in the proper grouping and contrasts, and this process will serve as a general test of their accuracy, and apply them to their most important purpose at the same time.

Some of the more disastrous periods of mortality of the summer have been attributed directly to extreme temperature, while the attendant condition of humidity has scarcely been recognized as influencing results. Violent epidemics, and even contagious diseases, may have been initiated by some maximum of these conditions, and the subsequent propagation and continuation may be due to other causes more difficult to decide upon. Without assuming entire dependence upon the conditions of climate we now measure for epidemics of the more violent character, it is still a point gained of unusual importance when numerical values may be given to humidity as well as temperature. With these the statistics of disease and mortality may ultimately undergo thorough comparison and examination, and to that research the greatest and final results will belong.

The climatic research conducted by the Smithsonian Institution now embraces every climatic district of the country. The full registers, including with the more common observations the difficult one of the temperature of evaporation, from which is deduced the relative humidity, number about thirty, which are immediately returned at the close of an observed month. There are others in the New York and Military System, accessible at a later period; but the first suffice to determine every important district in this respect. Correct temperature observations for the open air are reported in the same prompt manner from about two hundred stations, of which about fifty measure carefully the amount of precipitation.

From these data the present exhibit is prepared. It will present, first, the several periods of excessive heat in the three summer months of the present year, and the comparison of various districts during those periods.

Second, the periods of excessive humidity, and of unusual amount of precipitation; and the relation of these in time and place to the division of excessive variations of temperature.

It also marks extremes of absence of humidity, and of cold, which are less important as sanitary conditions standing alone, though the low humidity apparently occurs generally as a very important modification of the injurious character of a period of excessive heat.

The first general high temperature of the season occurred on the 3d to the 5th of June, extending from Montreal to Florida, but this did not occur at the West generally. At the South it was a day later than in New York, and its maximum at the North, from Montreal to New York, 83°, and from Chapel Hill, N. C., to Savannah, Ga., 92°. The period was so short that on general consequence would probably attach to it, however, and the amount of humidity was not unusual.

From the 14th to the 18th, the heat was excessive and general. At the extreme West, in Minnesota, Iowa and Wisconsin, it occurred on the 12th and 13th; the atmosphere affected passing eastward across the continent as a well-defined body, according to what seems a law of all dynamic or inconstant climatic phenomena. It did not extend beyond Camden, S. C., southward, and attained a higher point at the West than elsewhere, of 90° to 94° in Ohio, Kentucky, and westward in the same latitudes. Succeeding this, a general excess of temperature occurred from the 20th to the 23d, less than the preceding in the extreme North, and with a considerable fall there on the 22d, but quite unusual and long-continued at almost every other part of the country. The maxima varied from the 20th to 23d, and ranged from 90° to 97°. With the slight allowance for excess, which usually should be made in the readings at these extreme points, the maximum of 95° was probably general, from New York to Savannah, on the 23d, and the same degree the day previous in Ohio and Michigan. In Tennessee and south-westward, the same point was reached on the 20th; in Mississippi and Texas still one day earlier.

Lastly, a most extraordinary extreme of heat occurred on the 29th and 30th; in this case, as before, beginning earliest at the West by a day and a half for the distance from St. Louis to Washington. This extreme was central in the latitude of Washington, and was limited at Savannah on the South, and Burlington, Vt., on the North. It attained 96° to 98° in Tennessee, Kentucky, and Southern Ohio, and 99.5° to 102° at Washington and in Eastern Virginia and N. Carolina. This is without any known parallel in the records of temperature here, and is several degrees above any recorded temperatures at New Orleans, Mobile, or Savannah.

The general temperature of July was also high, and slightly above the normal mean in most parts of the United States. The mean of June was much above the normal one, attaining a maximum of excess in Wisconsin and Illinois of nine degrees. It was there 4.5° above the mean for July,—this excess being less at the east, where the two months were nearly equal, and June 5° above the normal mean.

The excessive heat of the last days of June, was prolonged through the 1st and 2d of July at 94° in Virginia and at the South, and the range was generally high at Philadelphia and South until the 10th, when it was again at 92 to 84°. North of latitude 40° there were no great heats in this month, and the temperature was quite uniform throughout. The temperature was at or above 90°, after the middle of the month, only in Central Georgia and Alabama, and westward in this latitude to Texas, for two or three days about the 20th, and again at the close of the month.

In August, a period of general excessive heat occurred, beginning, as usual, earlier at the West, and reaching 90° at Lac-qui-parle, on the St. Peter's River, Minnesota, (lat. 45°, long. 96°,) on the 7th and 8th. The maximum in Illinois and the adjacent States was 90° to 94° from the 8th to the 13th, in Ohio and Kentucky nearly the same; and, passing eastward a little later through Pennsylvania, the district of greatest excess was central at New York from the 12th to the 14th.

The temperature was below 80° at Cedar Keys, Talahassee, and Pensacola, Fla., through these days, and at no place South reached 90°. Later in the month, from the 25th to the 31st, the heat was unusually great in the South-west,—Texas, the Cherokee Territory, and Mississippi,—with an extraordinary reverse in Iowa and the adjoining States. Frosts occurred in all the States West and North of Ohio at some day from the 25th to 28th inclusive; and in some parts of Iowa, Wisconsin, and Michigan, there were two or three in succession, and great injury was done to vegetation. At Lac-qui-parle,

Minnesota, 3° farther North, and nearly a thousand feet higher, there was one very slight frost on the 27th.

These extraordinary conditions of temperature become more important when connected with the accompanying conditions of humidity. As general comparisons of this last condition have hardly been possible, heretofore, the present summer cannot be compared with previous years, except in one or two extremes of its sanatary effect.

The observations of humidity are, however, believed to be of the very best character. The temperature of the *natural evaporation* has been taken under uniform directions, and with uniform instruments. The actual *evaporating effect* of the air at the time, as the measure of its true evaporating power in a climatic sense, and such as could affect animal life and the growth of vegetation, has been sought in the directions given, and in the practice of the observers.

From such observations the fraction of saturation, or the percentage of humidity existing in the air at the time, has been deduced by Regnault's formulas, first determined theoretically from the latent and specific heat of air and aqueous vapor, and subsequently modified in the numerical elements to conform to the results derived from actual experiment.

These results are arranged in a tabular form for greater convenience of comparison. The "relative humidity" is the percentage of moisture which the air at the time contains. 100 being full saturation. The absorbent power of the air in highly heated periods is directly as this decrease of the degree of saturation, and is the most precise measure possible of the sanatary condition which is peculiarly climatic, and not dependent on miasmatic or other secondary causes.

A brief reference to the prominent points in this comparison may assist the examination. It may be stated that the fractions of saturation are much more variable in cold than in warm climates at all temperatures. The mean summer saturation at New Orleans, by the valuable observations of Dr. Barton,* is .857, and the mean saturation for the year .811. The degree here is quite constant through successive years for the different seasons, and has its maximum in summer. The maximum in the northern portion of the United States is also in summer, but the mean is something more than one-tenth of saturation less than that at New Orleans.

The fractions given in the tabular statements used for the present general comparisons are from one observation only for the day, that at 2, P. M.; which is very near the hour for maximum temperature also. It would be equally important to give the mean of the day, but as the labor would be very much increased, the single hour of extreme range of the several conditions, giving the most striking contrasts, has been thought sufficient for the present purpose.

The heats of June are seen at once to have been remarkably dry. The fraction of saturation was at a mean of about 50 in the North-east, and but 40 to 45 in the interior and in Texas, during the hot days from the 14th to the 17th, though much higher at Pensacola and the extreme South, where the heat was not so great. At the 20th to the 23d, the rate was about the

---

**Report to the La. State Med. Soc., on the Meteorology, Vital Statistics, and Hygiene of Louisiana.* By Dr. E. H. BARTON, 1851. The amounts here given are a summary from observations at sunrise, midday and 9, P. M., for eight years.

same in the districts of excessive heat. On the 29th and 30th, the percentage was but 35 to 40 in the narrow district through Tennessee, Kentucky, and Virginia, which marked 100° as the maximum temperature. The coincidence of unusual absence of humidity with this unequaled temperature was fortunate. The usual saturation with this degree of heat, would have been extremely destructive to health and life.

The first two days of July were a continuation of the conditions of the last of June. The remainder of the month was not unusual in its hygrometric character generally, though at New Orleans, where, unfortunately, we have no observations after May, the evidences of high saturation are given in the profuse and constant rains of the middle of the day, preceded by a hot and oppressive morning. This weather was reported as quite uniform for the first and most fearfully fatal month of the yellow fever. The approach to a tropical character in the midsummer saturation and daily rains, at New Orleans, is more or less distinctly marked in different years at all times, but the present summer seems to have attained a maximum in this respect, and to have given in this way terrible effect to the epidemic, when once induced, in whatever manner that may have been. It is hoped that careful observations of the humidity of the yellow fever period of the present summer there, have been made. The Gulf Coast in Texas and Florida does not properly represent the humidity or other climatic peculiarities of New Orleans, nor does any place in the interior from which we have observations.

The great heat of August was most remarkable in its hygrometric condition also, and universally attended with a high fraction of saturation. At Washington it was 55 to 60, and at New York near 70 per cent. at 2, P. M., and almost at saturation morning and evening. Though the temperature of the air was but 90° to 94°, or 6° less than that of the last of June in Washington and Richmond, where no fatal effects were felt, the mortality from the effect of great heat with great saturation was frightful. By reference to the tabular statements it may be seen that the absolute degree of heat could not have caused the fatality alone, or without the attendant humidity. Some term more specific or more comprehensive than *sun-stroke* seems, therefore, required to designate the fatal congestion, or whatever may be the immediate cause of death in these cases.

Another, and perhaps clearer point for comparison, is the *temperature of evaporation,* or that of the wet-bulb thermometer alone. The dew-point has usually been obtained for such comparisons, but it is far less easy to obtain without error; and, as it is itself no absolute determination, but an intermediate point, only, it is quite generally discontinued.

The temperature of evaporation at New York, at the time of greatest mortality in August, was from 80° to 84°, being higher than the maximum temperature of evaporation at New Orleans at any time in 1852 by two degrees. At the latter place it reached 82° but once in that year. At Austin, Texas, for the last August, the same maximum was 78°; at Savannah, Ga., 79°; at Camden, S. C., Culloden, Ga., and Washington, 77°. Jacksonville, Fla., and Eutaw, in Central Alabama, were the only places at the South giving a temperature of evaporation of 84°, or as high as that at New York on the 13th and 14th of August.

With such conditions of temperature and saturation, in a nominally elastic and cool climate, it is not wonderful that the mortality from its immediate effect should have been very great. It is more remarkable that some incipient epidemic was not started in full vigor to rage after the principal inducing cause had passed away. With the exceptions of New Orleans, and New York at this limited period, the heats of the summer, though extreme, have been attended with a low humidity, and have been unusually favorable in this respect. The maximum heat of June, which was from 4° to 9° above the mean, could scarcely have passed without most injurious effects at the ordinary percentage of humidity. The excessive heats of lower Texas, the Rio Grande Valley, and other districts, where the thermometer rises to 112 and 115°, have a temperature of evaporation not above that at New Orleans, with the air at 87°. At Austin, Texas, with the air at 98° several times in June, the temperature of evaporation never rose above 78°, and at the highest air temperature was at 74° and 76°,—or nearly 10° below the temperature of evaporation at New York, when the air thermometer did not exceed 95°.

The heats of those districts are therefore endurable and even pleasant, at a degree which would seem fatal to life, from the great evaporating power and elasticity of the atmosphere which uniformly prevails.

As an attendant condition resulting from humidity, the amount of rain for the three summer months should be given in this connection.

The normal distribution of rain among all the months is very nearly equal in the North-eastern parts of the United States. The amount varies through a wide range, however, in different years. Each of the summer months may change from an entire absence of rain to an ordinary maximum of twelve inches; and, in one or two instances, an extreme of twenty to twenty-two inches. It is difficult, therefore, to say as definitely what the relation of either month has been to the normal condition from this observation, as from either temperature or the hygrometric observation.

In June, the amount of rain was much less than usual, generally. In Maine and most of New England, the amount was least, varying from half an inch to an inch and a half in depth. The amount for all central parts of the United States was within these limits, except the eastern portion of Virginia. In Florida, there was about the usual amount (of 3 to 3½ inches) In a small district central in Iowa it reached 6½ inches.

In July, the amount was particularly large at Philadelphia and Southward to Florida, where it was 11.5 inches. In Alabama and at New Orleans, the amount was nearly as great, and in Iowa and Wisconsin it was again large, —from 6 to 8 inches. In other parts of the United States and Canada, the usual amount of from 2.5 to 4 inches fell. There were, however, large districts in which the least of these amounts, in conjunction with the very little rain in June, caused very severe droughts. The principal of these was in Eastern Ohio, and Western Pennsylvania, and New York. In Central New York the amount was variable and the drought in some places severe.

In August, the rains were excessive in a district extending from the lower part of New Hampshire to Northern New Jersey; at Bloomfield, N. J., and New York, the amount falling was near 12 inches. From Baltimore to Savannah, also, the amount was large, being from 5 to 6.5 inches, and about 1½ inches more than usual. The last days of July and the first days of August, gave an excessive precipitation in Eastern Pennsylvrnia and New York, New Jersey, &c. These flooding rains, which gave in some instances 4 to 8*

* Register of R. L. Cooke, Principal of Bloomfield Academy, New Jersey.

inches in depth of water in a single storm of a few hours, attended very warm weather, and immediately preceded the fatal heats of 12th to 14th August.

They were also followed by profuse rains, and the whole period from 25th July to 15th of August, seemed a substitution of a tropical climate for the usually elastic one, in the district referred to.

The tabular statistics given with this paper are intended to embrace the more marked extremes at the best observed stations of temperature of the air and of evaporation, with the humidity as determined from these. For June, thirty-two stations are given with these observations. For July, the general reference to extremes, and the table of mean temperatures and amount of rain, appear sufficient. The conditions were general, and the extremes not signally marked in most parts of the United States. At New Orleans there were evidently unusual features, but the districts adjacent to it do not sufficiently represent them to warrant any tabular arrangement of extracts. It is to be regretted that we have not observations there, though they may have been made for part of the month, and may ultimately be compared with other districts.

For August, extracts are given from the registers at twenty-four stations, sufficient to represent every important district.

Mean temperatures and amount of rain are given in a tabular form from over ninety stations.

The temperatures were observed at 7, A. M., 2, P. M., and 9, P. M., in nearly every case; the exceptions are two or three instances, in which the hours were 6, A. M., 2, P. M., and 10, P. M., and as many at sunrise, 9, 3, 9; but in these instances the mean does not differ essentially from the first. Some slight discrepancies appear in this statement, though the general correspondence of stations which should agree is quite apparent. In reference to these means the comparison of June and July with each other and with the normal mean has already been made. The mutual comparison of the three months shows an extraordinary uniformity in absolute position. This seems, at first, to give July a considerable fall below the normal mean, but the truth is rather that it is itself slightly above, and June and August very much above the mean of other years. The normal curve at New York and Northward is a rise of 4° to 4.5° from the mean for June to that for July, and a fall of 3° to that of August. At Philadelphia and Southward, it is somewhat less, decreasing to the Gulf Coast, where the curvature nearly disappears. Fifty-seven consecutive years of observation near Philadelphia, give 4.3° as the first value, or the change from June to July, and 2.9° as the second, or fall to the mean of August. With this general guide, the comparison of the various stations in this respect may readily be made by the reader.

The amount of rain may be compared with normal amounts in this general way also, in addition to the references already made to the extremes. In the New England States the observed mean precipitation for the last twenty years is greatest in August, and least in July, of the three summer months. Three and one-fourth, two and one-fourth, and three and three-fourths inches, consecutively represent the summer fall of rain there for the three months.

In New York this difference disappears. There and Southward to Baltimore the mean of long series of observations gives about 3.5 inches as the mean for each summer month.

At the South the amount is greater, with a tendency to excess in August in the Atlantic States, but elsewhere the summer months are nearly uniform in quantity, and the amount greater than in other parts of the year. Six to seven inches of rain in the interior at the South, or away from the mountains or the immediate coast, is nearly the general mean for each summer month.

At Cincinnati, the months again become nearly equal in amount of rain, through the year, and the summer quantity, monthly, 4.4 inches. In Iowa it is more, though very irregular; and elsewhere at the North-west falls off rapidly, preserving a relation for each month of about one-tenth to the annual amount. This hasty reference to mean quantities cannot be made very clear here, with the space assigned and the present purpose, but the few points given may assist in judging of profusion or failure of rain for the summer in the general way required for sanatary comparison.

In conclusion, it may be said that a more precise definition of periods of time, and a closer limitation of districts, in respect to each branch of the subject, is very desirable, and is possible, also, from the data already at hand, with greater space, and time for a more full elaboration and comparison. Limited districts, of extreme drought in these months, and others of the spring, have occurred. Some of these, in time and place, would be quite important to define in the special purpose we now have, and still more in their connection with the agricultural prosperity of the country for the year.

That our climate has general characteristics, in both the conditions treated here, which permit a clear grouping and statement almost at the time the events are passing, is considered fully sustained by these references and statistics. This is itself of great interest, as showing that we have the means at hand to make the research almost coincident in time with the phenomena. The great advantage of this in all that pertains to the sanatary condition will be recognized at once.

### JUNE, 1853.

BURLINGTON, VT.—Rev. Z. Thompson.

| | *Air.* | *Evap'n.* | *Humidity.* |
|---|---|---|---|
| 14th | 91. | 76. | .47 |
| 15th | 92. | 75. | .39 |
| 16th | 82. | 74. | .67 |
| 17th | 85. | 74. | .58 |
| 20th | 91. | 78. | .53 |
| 21st | 82. | 65. | .35 |
| 22d | 79. | 67. | .50 |
| 23d | 92. | 76. | .46 |
| 30th | 89. | 75. | .50 |

PITTSFIELD, MASS.—J. H. Benjamin.

| | | | |
|---|---|---|---|
| 14th | 83.3 | 69.8 | .49 |
| 15th | 85. | 71.6 | .50 |
| 16th | 85. | 75. | .62 |
| 17th | 75. | 70. | .78 |
| 29th | 74.3 | 68.9 | .74 |
| 30th | 80.9 | 73. | .67 |

WORCESTER, MASS.—Dr. E. A. Smith.

| | | | |
|---|---|---|---|
| 14th | 82.4 | 66.2 | .39 |
| 15th | 86. | 66.2 | .30 |

WORCESTER, MASS.—(Continued.)

| | *Air.* | *Evap'n.* | *Humidity.* |
|---|---|---|---|
| 16th | 87. | 69.8 | .39 |
| 17th | 80.6 | 68. | .49 |
| 20th | 86.9 | 71.6 | .45 |
| 21st | 90. | 71.6 | .37 |
| 29th | 73.4 | 68. | .74 |
| 30th | 84.5 | 71.6 | .51 |

S. MARTIN'S, MONT'L.—Dr. C. Smallwood.

| | | | |
|---|---|---|---|
| 14th | 91.3 | 78.6 | .53 |
| 15th | 94.3 | 79.6 | .50 |
| 16th | 97. | 79.9 | .56 |
| 17th | 83. | 72.8 | .61 |
| 20th | 86.7 | 77. | .65 |
| 21st | 72. | 61. | .50 |
| 22d | 61. | 54.8 | .66 |
| 23d | 90.6 | 80. | .62 |
| 30th | 82. | 70. | .55 |

AMHERST, MASS.—Prof. E. S. Snell.

| | | | |
|---|---|---|---|
| 14th | 86. | 70.7 | .43 |
| 15th | 88. | 77. | .58 |

AMHERST, MASS.—(Continued.)

| | *Air.* | *Evap'n.* | *Humidity.* |
|---|---|---|---|
| 16th | 89.5 | 77.7 | .58 |
| 17th | 79. | 74. | .77 |
| 20th | 89. | 76.3 | .52 |
| 21st | 91.3 | 76.8 | .49 |
| 22d | 85. | 79. | .79 |
| 23d | 81.5 | 75.9 | .74 |
| 29th | 73.6 | 70.7 | .86 |
| 30th | 86.5 | 78.4 | .68 |

NEWBURYPORT, MASS.—Dr. H. C. Perkins.

| | | | |
|---|---|---|---|
| 14th | 72.5 | 68.7 | .71 |
| 15th | 83.3 | 63.5 | .55 |
| 16th | 95. | 68.9 | .49 |
| 17th | 85. | 68.9 | .57 |
| 20th | 87.6 | 76.1 | .56 |
| 21st | 80.7 | 69.4 | .54 |
| 29th | 69.6 | 63.5 | .59 |
| 30th | 78.8 | 63.5 | .71 |

POMFRET, CT.—Daniel Hunt.

| | | | |
|---|---|---|---|
| 13th | 70. | 65. | .75 |
| 14th | 82. | 74. | .67 |
| 15th | 78. | 74. | .82 |
| 16th | 82. | 76. | .75 |
| 17th | 77. | 73. | .82 |
| 20th | 86. | 77. | .65 |
| 21st | 89. | 80. | .66 |
| 22d | 86. | 81. | .79 |
| 29th | 72. | 69. | .86 |
| 30th | 84. | 78. | .75 |

MADRID, N. Y.—E. A. Dayton.

| | | | |
|---|---|---|---|
| 13th | — | — | — |
| 14th | 87.4 | 73.4 | .55 |
| 15th | 91.4 | 73. | .47 |
| 16th | 91.2 | 76.6 | .55 |
| 17th | 79.5 | 68. | .58 |
| 20th | 84.3 | — | — |
| 21st | 72.3 | 59. | .49 |
| 22d | 74.8 | 65.1 | .62 |
| 29th | 79.7 | 73.4 | .45 |
| 30th | 83.1 | 65.5 | .45 |

BLOOMFIELD, N. J.—R. L. Cooke.

| | | | |
|---|---|---|---|
| 13th | 82.5 | 71. | .54 |
| 14th | 86.5 | 74. | .54 |
| 15th | 84. | 72. | .54 |
| 16th | 84. | 75. | .63 |
| 17th | 57.5 | 77. | .60 |
| 20th | 96. | 82. | .53 |
| 21st | 99.5 | 83. | .48 |
| 22d | 95. | 81. | .51 |
| 29th | 76. | 71.5 | .80 |
| 30th | 89. | 84. | .80 |

PHILADELPHIA.—Prof. J. A. Kirkpatrick.

| | *Air.* | *Evap'n.* | *Humidity.* |
|---|---|---|---|
| 13th | 79. | 68. | .55 |
| 14th | 87. | 76. | .59 |
| 15th | 87. | 74. | .52 |
| 16th | 87. | 73. | .49 |
| 17th | 85. | 75. | — |
| 20th | 96. | 79. | .45 |
| 21st | 95. | 80. | .50 |
| 22d | 95. | 79. | .46 |
| 29th | 85. | 76. | .64 |
| 30th | 95. | 80. | .50 |

LIMA, PA.—Joseph Edwards.

| | | | |
|---|---|---|---|
| 13th | 75.7 | 67.4 | .63 |
| 14th | 84.5 | 75.2 | .63 |
| 15th | 84.7 | 71.8 | .51 |
| 16th | 81.8 | 70.8 | .56 |
| 17th | 71.2 | 69. | .89 |
| 20th | 87.8 | 73.7 | .49 |
| 21st | 90.8 | 77.9 | .53 |
| 22d | 90.3 | 78. | .56 |
| 29th | 82.4 | 77. | .77 |
| 30th | 90.7 | 77.9 | .54 |

NORRISTOWN, PA.—Rev. J. G. Ralston.

| | | | |
|---|---|---|---|
| 13th | 81. | 70. | .56 |
| 14th | 89. | 78. | .59 |
| 15th | 87. | 72. | .45 |
| 16th | 87. | 72. | .45 |
| 17th | 72. | 72. | 1.00 |
| 20th | 90. | 76. | .50 |
| 21st | 93. | 81. | .58 |
| 22d | 93. | 77. | .46 |
| 29th | 87. | 78. | .65 |
| 30th | 90. | 77. | .56 |

HOLLIDAYSBURG, PA.—I. R. Lowrie.

| | | | |
|---|---|---|---|
| 13th | 85.8 | 66.9 | .32 |
| 14th | 89.9 | 71.2 | .36 |
| 15th | 86.3 | 72.7 | .50 |
| 16th | 88.7 | 71.2 | .39 |
| 17th | 79.3 | 68. | .53 |
| 20th | 92.4 | 72.3 | .34 |
| 21st | 86.3 | 76.1 | .61 |
| 22d | 86.7 | 75.9 | .59 |
| 29th | 34.7 | 75.7 | .39 |
| 30th | 34.6 | 74.8 | .38 |

BALTIMORE, MD.—Dr. G. H. Steiner.

| | | | |
|---|---|---|---|
| 13th | 82. | 70.1 | .53 |
| 14th | 87.4 | 75.2 | .54 |
| 15th | 86.9 | 73. | .49 |
| 16th | 87. | 70.1 | .40 |
| 17th | 77.5 | 70.1 | .68 |
| 20th | 95.5 | 75.3 | .36 |

BALTIMORE, MD.—(Continued.)

| | Air. | Evap'n. | Humidity. |
|---|---|---|---|
| 21st | 95.7 | 75.5 | .36 |
| 22d | 96.2 | 78.8 | .44 |
| 29th | 88.1 | 78.9 | .35 |
| 30th | 92.3 | 73.5 | .38 |

ALEXANDRIA, VA.—Benj. Hallowell.

| | | | |
|---|---|---|---|
| 13th | 78.8 | 69.8 | .62 |
| 14th | 90.5 | 76.1 | .49 |
| 15th | 86. | 75.2 | .59 |
| 16th | 86.9 | 73.4 | .51 |
| 17th | 71.6 | 69 8 | .91 |
| 20th | 90.5 | 77. | .52 |
| 21st | 90.5 | 77. | .52 |
| 22d | 89.6 | 78.8 | .60 |
| 29th | 91.4 | 74.3 | .42 |
| 30th | 95. | 75.2 | .37 |

SAVANNAH, GA.—Dr. John F. Posey.

| | | | |
|---|---|---|---|
| 13th | 84. | 73.9 | .60 |
| 14th | 86. | 68.7 | .40 |
| 15th | 85.8 | 72.6 | .51 |
| 16th | 83.8 | 74.1 | .61 |
| 17th | 84. | 73.5 | .59 |
| 20th | 81.3 | 76.1 | .77 |
| 21st | 87.8 | 78.6 | .64 |
| 22d | 92.3 | 77.9 | .50 |
| 29th | 90.8 | 75.2 | .46 |
| 30th | 91.2 | 78.4 | .54 |

CAMDEN, S. C.—Thornton Carpenter.

| | | | |
|---|---|---|---|
| 13th | 85.9 | 70. | .35 |
| 14th | 92. | 72.2 | .35 |
| 15th | 93. | 73.7 | .37 |
| 16th | 91. | 73. | .39 |
| 17th | 89. | 75. | .50 |
| 20th | 78.5 | 73.8 | .79 |
| 21st | 89.5 | 78. | .58 |
| 22d | 93. | 75. | .40 |
| 29th | 97.5 | 73.6 | .27 |
| 30th | 97.6 | 74. | .29 |

SPARTA, GA.—Dr. E. M. Pendleton.

| | | | |
|---|---|---|---|
| 13th | 93. | 73. | .35 |
| 14th | 94. | 70. | .26 |
| 15th | 96. | 70. | — |
| 16th | 95. | 71. | .26 |
| 17th | 95. | 73. | .31 |
| 20th | 90. | 78. | .56 |
| 21st | 92. | 72. | .54 |
| 22d | — | — | — |
| 29th | 96. | 70. | — |
| 30th | 97. | 72. | .26 |

CULLODEN, GA.—John Darby.

| | Air. | Evap'n. | Humidity. |
|---|---|---|---|
| 13th | 85.1 | 68.7 | .40 |
| 14th | 85.6 | 67.1 | .33 |
| 15th | 88.7 | 69.8 | .35 |
| 16th | 86.7 | 67.1 | .30 |
| 17th | 88.5 | 70.8 | .38 |
| 20th | 79.5 | 72.3 | .69 |
| 21st | 88.5 | 75.2 | .50 |
| 22d | 87.8 | 75.9 | .55 |
| 29th | 93.2 | 73. | .34 |
| 30th | 92.5 | 72.3 | .34 |

EUTAW, ALA.—A. Winchell.

| | | | |
|---|---|---|---|
| 13th | 95. | 78. | .44 |
| 14th | 92. | 75. | .42 |
| 15th | 96. | 78. | .42 |
| 16th | 96. | 79. | .45 |
| 17th | 98. | 79. | .40 |
| 20th | 99. | 82. | .47 |
| 21st | 93. | 82. | .61 |
| 22d | 98. | 83. | .51 |
| 29th | 104. | 82. | .36 |
| 30th | 101. | 86. | .52 |

JACKSONVILLE, FLA.—Dr. A. S. Baldwin.

| | | | |
|---|---|---|---|
| 13th | 82. | 76. | .74 |
| 14th | 81. | 74. | .71 |
| 15th | 81. | 75. | .74 |
| 16th | 80. | 75. | .78 |
| 17th | 83. | 77. | .75 |
| 20th | 85. | 80. | .82 |
| 21st | 88. | 83. | .80 |
| 22d | 84. | — | — |
| 29th | 87. | 79. | .69 |
| 30th | 84. | 79. | .79 |

PENSACOLA, FLA.—Com. Tatnall.

| | | | |
|---|---|---|---|
| 13th | 81. | 78. | .87 |
| 14th | 81. | 77. | .83 |
| 15th | 82. | 76. | .74 |
| 16th | 82. | 79. | .87 |
| 17th | 82. | 78. | .83 |
| 20th | 83. | 78. | .79 |
| 21st | 77. | 76. | .95 |
| 22d | 84. | 78. | .75 |
| 29th | 86. | 83. | .88 |
| 30th | 85. | 79. | .76 |

AUSTIN, TEXAS.—Dr. Samuel K. Jennings.

| | | | |
|---|---|---|---|
| 10th | 96. | 72. | .27 |
| 11th | 98. | 74. | .28 |
| 12th | 83. | 73. | .60 |
| 13th | 84 | 72. | .54 |
| 14th | 90. | 76. | .50 |

AUSTIN, TEXAS.—(Continued.)

| | *Air.* | *Evap'n.* | *Humidity.* |
|---|---|---|---|
| 15th | 86. | 68. | .36 |
| 16th | 70. | 66. | .80 |
| 17th | 90. | 76. | .50 |
| 18th | 94. | 74. | .36 |
| 19th | 97. | 74. | .30 |
| 20th | 92. | 74. | .39 |
| 21st | 89. | 78. | .59 |
| 22d | 93. | 78. | .49 |
| 26th | 93. | 76. | .43 |
| 27th | 90. | 76. | .50 |
| 28th | 92. | 76. | .45 |
| 29th | 96. | 76. | .37 |
| 30th | 96. | 77. | .39 |

KNOXVILLE, TENN.—O. W. MORRIS.

| | | | |
|---|---|---|---|
| 5th | 89.9 | 73.2 | .41 |
| 6th | 88.1 | 71.2 | .40 |
| 12th | 96. | 69. | .38 |
| 13th | 86.2 | 66. | .30 |
| 14th | 88.8 | 68.9 | .32 |
| 15th | 90.1 | 70.5 | .34 |
| 16th | 87. | 70.1 | .40 |
| 28th | 91. | 72.3 | .37 |
| 29th | 93. | 71.9 | .26 |
| 30th | 94.4 | 74.3 | .35 |

LEBANON, TENN.—PROF. A. P. STEWART.

| | | | |
|---|---|---|---|
| 5th | 87.8 | 70.5 | .39 |
| 6th | 86.9 | 70.7 | .41 |
| 12th | 89.7 | — | — |
| 13th | 89.9 | — | — |
| 14th | 91. | — | — |
| 15th | 91.4 | — | — |
| 16th | 89.9 | — | — |
| 28th | 92.6 | 71.6 | .32 |
| 29th | 93.2 | 75.2 | .40 |
| 30th | 95.9 | 73.6 | .31 |

CLARKSVILLE, TENN.—W. M. STEWART.

| | | | |
|---|---|---|---|
| 4th | 86.7 | 76.4 | .59 |
| 5th | 88.1 | 71.2 | .41 |
| 6th | 88.7 | 72.5 | .42 |
| 11th | 84.9 | 73.4 | .56 |
| 12th | 87.4 | 68. | .33 |
| 13th | 87.4 | 69.8 | .38 |
| 14th | 87.4 | 70.1 | .39 |
| 19th | 87.2 | 68. | .33 |
| 20th | 90.1 | 74.1 | .44 |
| 27th | 90.1 | 72.5 | .39 |
| 28th | 91. | 70.5 | .32 |
| 29th | 92.5 | 74.8 | .41 |
| 30th | 92.8 | 74.5 | .40 |

OBERLIN, O.—PROF. I. H. FAIRCHILD.

| | | | |
|---|---|---|---|
| 13th | 90. | 77. | .53 |
| 14th | 89. | 78. | .59 |

OBERLIN, O.—(Continued.)

| | *Air.* | *Evap'n.* | *Humidity.* |
|---|---|---|---|
| 15th | 93. | 83. | .64 |
| 16th | 91. | 80. | .60 |
| 17th | 97. | 68. | .61 |
| 18th | 82. | 72. | .59 |
| 19th | 90. | 82. | .70 |
| 20th | 92. | 81. | .61 |
| 21st | 95. | 82. | .55 |

HILLSBOROUGH, O.—J. McD. MATTHEWS.

| | | | |
|---|---|---|---|
| 13th | 85 5 | 73. | .53 |
| 14th | 88.5 | 70. | .36 |
| 15th | 80.5 | 78. | .89 |
| 16th | 87.5 | 73. | .47 |
| 17th | 79.5 | 67. | .49 |
| 18th | 81. | 64. | .35 |
| 19th | 87. | 71. | .43 |
| 20th | 89.5 | 72. | .39 |
| 21st | 90. | 74. | .44 |
| 28th | 87.5 | 72. | .44 |
| 29th | 91.5 | 76. | .46 |
| 30th | 93. | 74. | .37 |

BROOKLYN, MICH.—DR. M. K. TAYLOR.

| | | | |
|---|---|---|---|
| 13th | 88. | 73. | .46 |
| 14th | 90. | 74. | .44 |
| 15th | 94. | 82. | .58 |
| 16th | 72. | 70. | .90 |
| 17th | — | — | — |
| 18th | 81. | 71. | .49 |
| 19th | 88. | 80. | .69 |
| 20th | 90. | 81. | .66 |
| 21st | 97. | 83. | .53 |
| 22d | 92. | 82. | .70 |
| 28th | 90. | 82. | .70 |
| 29th | 79. | 73. | .74 |
| 30th | 81. | 72. | .63 |

N. HARMONY, IND.—JOHN CHAPELSMITH.

| | | | |
|---|---|---|---|
| 10th | 90. | — | .37 |
| 11th | 91. | — | .37 |
| 12th | 90. | — | .41 |
| 13th | 92. | — | .39 |
| 14th | 93. | — | .37 |
| 15th | 92.5 | — | .44 |
| 16th | 89. | — | .54 |
| 17th | 86. | — | .34 |
| 18th | 86. | — | .52 |
| 19th | 92. | — | .39 |
| 20th | 95. | — | .60 |
| 21st | 91. | — | .60 |
| 22d | 94. | — | .52 |
| 23d | 76. | — | .56 |
| 24th | 78. | — | .44 |
| 25th | 81. | — | .38 |
| 26th | 89.5 | — | .34 |

### N. HARMONY, IND.—(Continued )

| | *Air.* | *Evap'n.* | *Humidity.* |
|---|---|---|---|
| 27th | 93. | — | .46 |
| 28th | 97.5 | — | .38 |
| 29th | 95. | — | .50 |
| 30th | 92. | — | .46 |

### ALTON, ILL.—Dr. John James.

| | | | |
|---|---|---|---|
| 10th | 82. | 80. | .92 |
| 11th | 83. | 76. | .71 |
| 12th | 85. | 80. | .79 |
| 13th | 83. | 76. | .71 |
| 14th | 85. | 77. | .68 |
| 15th | 78. | 78. | 1.00 |
| 16th | 80. | 74. | .73 |
| 17th | 79. | 73. | .74 |
| 18th | 81. | 75. | .75 |
| 19th | 86. | 84. | .92 |
| 20th | 86. | 80. | .77 |
| 21st | 86. | 80. | .77 |
| 28th | 92. | 82. | .64 |
| 29th | 82. | 76. | .74 |
| 30th | 85. | 78. | .72 |

### POULTNEY, IOWA.—Benj. F. Odell.

| | | | |
|---|---|---|---|
| 10th | 92. | 78. | .51 |
| 11th | 87. | 75. | .55 |
| 12th | 92. | 77. | .48 |

### POULTNEY, IOWA.—(Continued.)

| | *Air.* | *Evap'n.* | *Humidity.* |
|---|---|---|---|
| 13th | 96. | 81. | .50 |
| 14th | 93. | 78. | .44 |
| 15th | 88. | 74. | .49 |
| 16th | 80. | 69. | .55 |
| 17th | 79. | 70. | .62 |
| 18th | 90. | 78. | .56 |
| 19th | 94. | 81. | .55 |
| 20th | 97. | 81. | .48 |
| 21st | 96. | 80. | .47 |
| 29th | 81. | 72. | .63 |
| 30th | 84. | 71. | .50 |

### BELLE FONTAINE, WIS.—Thomas Gay.

| | | | |
|---|---|---|---|
| 12th | 91. | 76. | .48 |
| 13th | 90. | 77. | .53 |
| 14th | 91. | 82. | .67 |
| 15th | 88. | 76. | .55 |
| 16th | 74. | 70. | .81 |
| 17th | 79. | 69. | .58 |
| 18th | 89. | 79. | .62 |
| 19th | 85. | 76. | .64 |
| 20th | 87. | 71. | .73 |
| 21st | 92. | 78. | .51 |
| 29th | 79. | 69. | .58 |
| 30th | 80. | 68. | .52 |

### AUGUST, 1853.

### ST. MARTIN'S, MONT'L.—Dr. C. Smallwood.

| | | | |
|---|---|---|---|
| 10th | 92.4 | 74.4 | .46 |
| 11th | 95.4 | 77.1 | .44 |
| 12th | 80.1 | 76.5 | .82 |
| 13th | 86.3 | 76.3 | .61 |
| 14th | 81. | 72.1 | .64 |
| 15th | 88.6 | 74. | .42 |
| 16th | 89. | 76. | .53 |

### BURLINGTON, VT.—Rev. Z. Thompson.

| | | | |
|---|---|---|---|
| 10th | 86. | 74. | .55 |
| 11th | 92. | 77. | .48 |
| 12th | 91. | 79. | .57 |
| 13th | 91. | 80. | .60 |
| 14th | 74. | 69. | .76 |
| 15th | 85. | 72. | .51 |

### AMHERST, MASS.—Prof. E. S. Snell.

| | | | |
|---|---|---|---|
| 10th | 85.2 | 74.1 | .57 |
| 11th | 88.7 | 80.6 | .69 |
| 12th | 91.9 | 75.9 | .45 |
| 13th | — | — | — |
| 14th | 91.5 | 82. | .65 |
| 15th | 76.6 | 69.8 | .69 |

### PITTSFIELD, MASS.—T. H. Benjamin.

| | | | |
|---|---|---|---|
| 10th | 82.4 | 73.4 | .63 |
| 11th | 84.2 | 71.6 | .52 |
| 12th | 86.9 | 79.5 | .78 |
| 13th | 86. | 75.5 | .60 |
| 14th | 87. | 84.2 | .88 |
| 15th | 79. | 68.7 | .55 |

### WORCESTER, MASS.—Dr. E. A. Smith.

| | | | |
|---|---|---|---|
| 10th | 84.5 | 73.4 | .57 |
| 11th | 88.5 | 73.7 | .47 |
| 12th | 90.6 | 75.2 | .46 |
| 13th | 91.4 | 76.2 | .47 |
| 14th | 89.6 | 75.2 | .49 |
| 15th | 71.6 | 63.1 | .61 |

### NEWBURYPORT, MASS.—Dr. H. C. Perkins.

| | | | |
|---|---|---|---|
| 10th | 75.2 | 70.3 | .77 |
| 11th | 90.3 | 75.2 | .47 |
| 12th | 92.8 | 77.7 | .47 |
| 13th | 70.5 | 67.4 | .83 |
| 14th | 90.8 | 79.7 | .59 |
| 15th | 68. | 6[illegible] | .70 |

POMFRET, CT.—Daniel Hunt.

| | Air. | Evap'n. | Humidity. |
|---|---|---|---|
| 10th | 81. | 75. | .74 |
| 11th | 85. | 77. | .68 |
| 12th | 85. | 77. | .58 |
| 13th | 89. | 82. | .73 |
| 14th | 84. | 80. | .83 |
| 15th | 71. | 67. | .80 |

BLOOMFIELD, N. J.—R. L. Cooke.

| | | | |
|---|---|---|---|
| 9th | 90. | 78. | .56 |
| 10th | 86.5 | 72.5 | .49 |
| 11th | 86. | 81. | .79 |
| 12th | 95. | 81. | .52 |
| 13th | 98. | 83. | .51 |
| 14th | 91. | 82.5 | .69 |

NORRISTOWN, PA.—Rev. I. Grier Ralston.

| | | | |
|---|---|---|---|
| 10th | 84. | 76. | .68 |
| 11th | 89. | 77. | .56 |
| 12th | 90.5 | 77. | .52 |
| 13th | 89. | 81. | .70 |
| 14th | — | — | — |
| 15th | 82.5 | 76. | .73 |

PHILADELPHIA, PA.—Prof. I. A. Kirkpatrick.

| | | | |
|---|---|---|---|
| 10th | 91. | 77. | .51 |
| 11th | 93. | 80. | .55 |
| 12th | 93.5 | 81. | .58 |
| 13th | 92. | 81. | .61 |
| 14th | 92.5 | 81. | .61 |
| 15th | 86. | 78. | .68 |

LIMA, PA.—Joseph Edwards.

| | | | |
|---|---|---|---|
| 10th | 86.3 | 77. | .64 |
| 11th | 88.1 | 77.9 | .60 |
| 12th | 88.7 | 80.9 | .67 |
| 13th | 87. | 80.6 | .74 |
| 14th | 87.2 | 80.6 | .73 |
| 15th | 82.4 | 77. | .77 |

HOLLIDAYSBURG, PA.—I. R. Lowrie.

| | | | |
|---|---|---|---|
| 10th | 86.1 | 72.6 | .50 |
| 11th | 87.2 | 72.8 | .48 |
| 12th | 89.6 | 73.9 | .45 |
| 13th | 90.3 | 75.3 | .47 |
| 14th | 88.7 | 77.1 | .57 |
| 15th | 87.8 | 75.3 | .54 |

BALTIMORE, MD.—Dr. S. H. Steiner.

| | Air. | Evap'n. | Humidity. |
|---|---|---|---|
| 10th | 92.6 | 72.5 | .34 |
| 11th | 92.6 | 79.7 | .54 |
| 12th | 93.9 | 77. | .44 |

BALTIMORE, MD.—(Continued.)

| | Air. | Evap'n. | Humidity. |
|---|---|---|---|
| 13th | 94.1 | 78.8 | .49 |
| 14th | 94.1 | 79.7 | .51 |
| 15th | 89.6 | 77.9 | .57 |

SMITHSONIAN INSTITUTION.

| | | | |
|---|---|---|---|
| 9th | 88.5 | — | — |
| 10th | 90.5 | — | — |
| 11th | 91.5 | — | — |
| 12th | 92. | — | — |
| 13th | 92. | — | — |
| 14th | 94. | — | — |
| 15th | 91. | — | — |

ALEXANDRIA, VA.—Benj. Hallowell.

| | | | |
|---|---|---|---|
| 10th | 85.1 | 75.2 | .62 |
| 11th | 87.8 | 75.2 | .53 |
| 12th | 87.8 | 77. | .59 |
| 13th | 88.7 | 77.9 | .59 |
| 14th | 89.6 | 77.9 | .57 |
| 15th | 92.3 | 77.9 | .50 |

CAMDEN, S. C.—Thornton Carpenter.

| | | | |
|---|---|---|---|
| 9th | 89.6 | 75.9 | .51 |
| 10th | 90.6 | 73. | .40 |
| 11th | 87.5 | 71. | .40 |
| 12th | 89.3 | 72.8 | .41 |
| 13th | 90.2 | 72. | .37 |
| 14th | — | — | — |
| 15th | 93.2 | 77.1 | .45 |

SAVANNAH, GA.—John F. Posey.

| | | | |
|---|---|---|---|
| 9th | 84.7 | 79.3 | .78 |
| 10th | 89.6 | 79.7 | .62 |
| 11th | 82.7 | 77. | .76 |
| 12th | 85.2 | 76.8 | .65 |
| 13th | 82.4 | 77.9 | .80 |
| 14th | 78.9 | 76.8 | .90 |
| 15th | 77. | 74.8 | .88 |
| 16th | 83.1 | 78.8 | .82 |

JACKSONVILLE, FLA.—Dr. A. S. Baldwin.

| | | | |
|---|---|---|---|
| 10th | 87. | 80. | .72 |
| 11th | 86. | 79. | .72 |
| 12th | 87. | 80. | .72 |
| 13th | 87.5 | 80. | .705 |
| 14th | 89. | 83. | .76 |
| 15th | 87. | 79. | .69 |

CULLODEN, GA.—John Darby.

| | | | |
|---|---|---|---|
| 10th | 86. | 75.2 | .59 |
| 11th | 85.1 | 72.5 | .52 |
| 12th | 85.2 | 76.1 | .63 |
| 13th | 84.2 | 71.6 | .52 |

CULLODEN, GA.—Continued.)

| | Air. | Evap'n. | Humidity. |
|---|---|---|---|
| 14th | 85.2 | 73.7 | .56 |
| 15th | 82.4 | 77. | .77 |

EUTAW, ALA.—A. Winchell.

| | | | |
|---|---|---|---|
| 10th | 91. | 82. | .67 |
| 11th | 93. | 84. | .67 |
| 12th | 91. | 80. | .60 |
| 13th | 88. | 79. | .65 |
| 14th | 89. | 78. | .59 |
| 15th | 91. | 83. | .70 |

AUSTIN, TEXAS.—Dr. Samuel K. Jennings.

| | | | |
|---|---|---|---|
| 6th | 96. | 78. | .42 |
| 7th | 96. | 77. | .40 |
| 8th | 94. | 75. | .38 |
| 9th | 88. | 76. | .55 |
| 10th | 86. | 76. | .61 |
| 11th | 85. | 76. | .64 |
| 12th | 76. | 76. | 1. |
| 13th | 88. | 76. | .55 |
| 14th | 88. | 74. | .49 |
| 15th | 82. | 76. | .74 |
| 16th | 95. | 76. | .38 |
| 17th | 99. | 76. | .31 |
| 18th | 98. | 78. | .38 |
| 25th | 90. | 76. | .50 |
| 26th | 92. | 78. | .51 |
| 27th | 92. | 78. | .51 |

AUSTIN, TEXAS.—(Continued.)

| | Air. | Evap'n. | Humidity. |
|---|---|---|---|
| 28th | 90. | 76. | .50 |
| 29th | 88. | 74. | .49 |

HILLSBOROUGH, O.—J. McD. Matthews.

| | | | |
|---|---|---|---|
| 10th | 88. | 71. | .40 |
| 11th | 90. | 71. | .35 |
| 12th | 89.5 | 77. | .54 |
| 13th | 85. | 77. | .68 |
| 14th | 78. | 74. | .82 |
| 15th | 81. | 74. | .70 |
| 16th | 86. | 74. | .55 |

OBERLIN, O.—Prof. J. H. Fairchild.

| | | | |
|---|---|---|---|
| 10th | 74. | 70. | .81 |
| 11th | 76. | 65. | .52 |
| 12th | 70. | 60. | .52 |
| 13th | 77. | 64. | .46 |
| 14th | 81. | 68. | .48 |
| 15th | 86. | 77. | .65 |

POULTNEY, IOWA.—Benj. T. Odell.

| | | | |
|---|---|---|---|
| 9th | 95. | 82. | .55 |
| 10th | 97. | 84. | .56 |
| 11th | 94. | 80. | .52 |
| 12th | 92. | 80. | .58 |
| 13th | 83. | 75. | .68 |
| 14th | 86. | — | — |
| 15th | 84. | 76. | .68 |

MEAN TEMPERATURE AND PRECIPITATION MONTHLY FOR THE SUMMER OF 1853, AT VARIOUS STATIONS IN THE UNITED STATES AND CANADA.

| STATIONS. | | MEAN TEMPERATURE. | | | AMOUNT OF RAIN. | | |
|---|---|---|---|---|---|---|---|
| | | June. | July. | Aug. | June. | July. | Aug. |
| N. S. | Albion Mines, | 61.6 | 69.1 | 65.0 | 3.105 | 2.948 | 4.120 |
| Me. | Bucksport, | 66.0 | 72.0 | .... | 0.050 | 3.400 | ...... |
| " | Biddeford, | 69.4 | 73.6 | 70.2 | 0.648 | 1.892 | 3.336 |
| " | Fryeburg, | .... | .... | .... | 0.830 | 1.580 | 3.125 |
| N. H. | Londonderry, | 67.0 | 70.0 | 68.4 | 1.250 | 2.980 | 6.800 |
| " | Manchester, | 69.5 | 72.5 | 69.2 | 1.061 | 1.986 | 9.154 |
| " | Concord, | 69.3 | 71.5 | 68.6 | 1.375 | 4.080 | 4.875 |
| Vt. | St. Johnsbury, | 65.0 | 65.9 | .... | 1.810 | 3.140 | ...... |
| " | Burlington, | 69.2 | 70.5 | 69.8 | 1.740 | 3.120 | 3.460 |
| C. E. | Montreal, | 68.7 | 68.0 | 69.1 | 3.131 | 3.102 | 7.089 |
| Mass. | Amherst, | 67.0 | 68.4 | 69.3 | 2.636 | 3.585 | 6.126 |
| " | Worcester, | 68.6 | 71.7 | 68.7 | 1.010 | 3.290 | 10.710 |
| " | Richmond, | .... | .... | .... | 2.375 | 4.000 | 10.225 |
| " | Newburyport, | 67.0 | 71.5 | 67.2 | 0.656 | 1.754 | 6.959 |
| " | N. Attleboro, | 67.4 | .... | .... | 1.840 | 2.022 | 4.250 |
| " | Barnstable, | 66.7 | 71.0 | 71.0 | 2.075 | 3.510 | 2.460 |
| Conn. | Pomfret, | 66.1 | 68.6 | 68.7 | 1.440 | 3.850 | 6.850 |
| " | Salisbury, | .... | .... | 65.1 | ...... | ...... | 3.666 |
| " | New London, | 66.7 | 70.4 | 72.1 | ...... | ...... | ...... |
| N. Y. | Fort Hamilton, | 69.0 | 73.5 | 74.2 | 4.420 | 5.830 | 5.500 |
| " | Fort Columbus, | 71.2 | 72.7 | 73.5 | 4.800 | 4.400 | 5.230 |
| " | West Point, | 69.1 | 70.8 | 71.4 | 3.770 | 10.480 | 7.800 |
| N. J. | Bloomfield, | 69.9 | 71.3 | 71.8 | 2.750 | 3.652 | 12.117 |
| N. Y. | Madrid, | .... | 68.5 | .... | 0.990 | 1.790 | ...... |
| " | Houseville, | .... | .... | 70.4 | ...... | 2.470 | 2.130 |
| " | Constableville, | .... | 69.2 | .... | 2.875 | 3.625 | ...... |
| " | Gouverneur, | 69.8 | 70.7 | .. | 1.650 | 0.800 | ...... |
| " | Lodi, | 69.5 | 69.5 | 70.0 | 2.250 | 0.750 | 4.750 |
| " | Buffalo, | 68.2 | .... | 70.1 | 1.000 | ...... | 2.150 |
| Pa. | Moss Grove, | 71.5 | 68.8 | 68.5 | 2.500 | 0.700 | 3.100 |
| " | Pittsburg, | 74.0 | 72.0 | 71.0 | 1.853 | 3.428 | 7.747 |
| " | Bedford, | 72.5 | 73.3 | 72.6 | 0.328 | 1.513 | 2.364 |
| " | Hollidaysburg, | 72.5 | 73.4 | 69.7 | 1.043 | 1.188 | 5.041 |
| " | Gettysburg, | 74.9 | 74.7 | 73.0 | 0.266 | 5.672 | 3.382 |
| " | Norristown, | 73.2 | 73.2 | 73.1 | 1.500 | 4.841 | 5.020 |
| " | Harrisburg, | .... | 79.6 | 78.1 | 0.592 | 3.687 | 2.853 |
| " | Morrisville, | 70.3 | 71.9 | 71.6 | 3.200 | 7.000 | 4.600 |
| " | Philadelphia, | 75.3 | 76.6 | 75.7 | 1.050 | 6.290 | 3.080 |
| " | Lima, | 71.5 | 72.9 | 71.2 | 1.065 | 6.890 | 2.960 |
| Md. | Baltimore, | 77.7 | 78.5 | 77.9 | 0.150 | 2.491 | 4.725 |
| " | Frederick, | 77.2 | 77.2 | 75.3 | 0.389 | 4.141 | 4.059 |
| " | Schellman Hall, | 74.0 | 74.2 | 77.2 | ...... | 2.625 | 6.625 |
| " | Washington, | 75.0 | .... | 77.5 | 2.353 | 5.346 | 4.555 |
| Va. | Alexandria, | 76.0 | 76.4 | 76.0 | 2.872 | 5.108 | 5.992 |
| " | Buffalo, | 77.0 | 75.1 | 75.7 | 0.638 | 3.899 | 2.733 |
| " | Lewisburg, | 74.0 | 75.0 | 75.5 | 2.300 | 4.400 | 2.450 |
| " | Bruns. Co., | .... | .... | .... | 2 600 | 5.800 | 4.000 |
| " | Portsmouth, | 79.6 | .... | .... | 0.390 | ...... | ...... |
| S. C. | Camden, | 79.3 | 80.3 | 78.6 | 3.312 | 7.163 | 5.763 |
| Ga. | Savannah, | 79.0 | 81.5 | 79.4 | 0.787 | 6.464 | 8.168 |
| " | Whitemarsh, Id., | .... | 80.9 | 78.6 | 1.280 | 5.280 | 5.580 |
| " | Sparta, | 81.6 | 82.8 | .... | 0.710 | 5.350 | ...... |
| " | Culloden, | 81.7 | 81.6 | 80.4 | 0.370 | 9.810 | 1.180 |

MEAN TEMPERATURE AND PRECIPITATION MONTHLY.—*Continued.*

| STATIONS. | | MEAN TEMPERATURE. | | | AMOUNT OF RAIN. | | |
|---|---|---|---|---|---|---|---|
| | | June. | July. | Aug. | June. | July. | Aug. |
| Ga. | Penfield, | .... | 77.8 | 76.7 | 0.228 | 3.720 | 1.900 |
| Fla. | Jacksonville, | 78.9 | 81.7 | 82.5 | 3.240 | 7.400 | 2.700 |
| " | Knox Hill, | 78.7 | 79.9 | 79.9 | 3.454 | 7.519 | ...... |
| " | Ced r Keys, | 80.1 | 83.2 | 81.9 | 3 262 | 11.475 | 3.625 |
| " | Talahassee, | 80.6 | 80.0 | 81.0 | 2.820 | 7.920 | 4.670 |
| " | Pensacola, | 80.0 | 82.1 | 82.4 | 1.262 | 2.512 | 1.566 |
| Ala. | Eutaw, | 83.7 | 81.4 | 81 2 | 0.780 | 8.933 | 5.806 |
| Tex. | Austin, | 80.8 | 82.0 | 81.0 | ...... | ...... | ...... |
| Tenn. | Knoxville, | 75.3 | .... | .... | 1.409 | ...... | ...... |
| " | Glenwood, | 76.4 | 75 4 | 74.5 | 0.900 | 3.671 | 4.525 |
| " | Lebanon, | 79.5 | 77.7 | .... | 1.052 | 4.987 | ...... |
| Ky. | Danville, | 79.3 | .... | 76.5 | 0.360 | ...... | 3.485 |
| " | Millersburg, | 78.1 | 75.6 | 74.9 | ...... | ...... | ...... |
| Ohio. | Cincinnati, | .... | 75.6 | .... | ...... | 4.810 | ...... |
| " | Marietta, | 75.0 | 73.1 | .... | 0.650 | 3.706 | 1.340 |
| " | Mount Vernon, | 78.9 | .... | .... | 2.359 | ...... | ...... |
| " | Granville, | 74.3 | ... | 72.6 | 1 300 | ...... | 4.060 |
| " | Zanesville, | 76 0 | 76.8 | 75.7 | ...... | 2.173 | ...... |
| " | Cleveland, | 68 5 | .... | .... | 3.417 | ...... | ...... |
| " | Oberlin, | 75.5 | 70.8 | 73.4 | 2 110 | 3.790 | 4.760 |
| Mich. | Detroit, | 76.5 | 75.0 | .... | 2.567 | 2.333 | ...... |
| " | Ann Arbor, | 71.4 | 69.6 | 72.2 | 0.710 | 1 100 | 3.960 |
| " | Brooklyn, | 73.6 | 71.2 | .... | 1.500 | 1.710 | ...... |
| " | Battle Creek, | 74.3 | 71.5 | 73.5 | 1.600 | 1 560 | 1.000 |
| Va. | Richmond, | .... | 73.5 | .... | 1.220 | 3.045 | 5.585 |
| Ill. | Athens, | 75.4 | 73.3 | 73.9 | 5.520 | 3.870 | 3.650 |
| " | Alton, | 75.7 | 74.3 | 74.9 | ...... | 3.102 | 2.304 |
| Ia. | Fort Madison, | 76.7 | 75.9 | 76.0 | 7.650 | 3.800 | 5.050 |
| " | Muscatine, | .... | 68.6 | 70.9 | 6.400 | 6.600 | 1.700 |
| " | Poulteney, | 70.5 | 68.8 | 68.6 | 3.304 | 6.325 | 0.520 |
| " | Bowen's Prairie, | 72.3 | 68.7 | 70.5 | ...... | 6.600 | 0.760 |
| Wis. | Platteville, | 74.4 | 72.3 | 74 9 | 3.625 | 7.750 | 1.125 |
| " | Janesville, | 73.2 | 69.1 | .... | 2.620 | 4.630 | ...... |
| " | Beloit, | 73 1 | 68.7 | 71.4 | 4.950 | 8.100 | ...... |
| Minn. | Lac-qui-parle, | 65.8 | 68.9 | 70.6 | ...... | 6.750 | ...... |
| Ind. | New Harmony, | 79.3 | 77.7 | .... | 1.230 | 2.430 | ...... |
| Wis. | Madison, | 70.1 | 67.8 | 70.3 | ...... | ...... | ...... |
| " | Emerald Grove, | 70.7 | 69.0 | 71.9 | ...... | ...... | ...... |
| " | Bellefontaine, | 71.4 | 70.0 | 71.3 | ...... | ...... | ...... |

—*New York Journal of Medicine for November.*

---

Dr. Wolf, in Berlin, had for several years the most satisfa tory results from the administration of the portio choparti, in hæmorrhage of the lungs, when it threatened to become dangerous. One or two tablespoonfuls are taken daily, until the hæmorrhage ceases. The portio choparti consists of balsam copavia, syrup tolutani, aqu. menth. and alcohol as ℥j; æther nit. gtt. xvi. M.—*Annalen des Charite, Krankenhauses,* 1852, No. 2.

